# The Effects of Cognitive–Perceptual Style on the Multilevel Organization of Postural Control During Motor Imagery and Immersive Virtual Reality

**DOI:** 10.3390/bs16101907

**Published:** 2026-10-15

**Authors:** Grigorii Iskarevskii, Elizaveta Vinogradova, Ivan Afanevich, Vladislav Ogarkov, Ekaterina Zyuzya, Margarita Nikulina, Artur Fedianin, Dmitry Onishchenko, Maxim Baltin

**Affiliations:** 1 Interdisciplinary Sports Research, Center for Genetics and Life Sciences, Sirius University of Science and Technology, 1 Olympic Ave., Sirius Federal Territory, Sochi 354340, Russia; iskarevskij.gv@talantiuspeh.ru (G.I.); ekaterina.zyuzya@mail.ru (E.Z.); nikulina.mi@talantiuspeh.ru (M.N.); onischenko.da@talantiuspeh.ru (D.O.); 2 Laboratory of Microbiome, Center for Life Sciences, National Laboratory Astana, Nazarbayev University, 53 Kabanbay Batyr Ave., Block S1, Astana Z05H0P9, Kazakhstan; 3 Research Institute of Physical Culture and Sports, Volga State University of Physical Culture, Sports and Tourism, 35 Universiade Village, Kazan 420010, Russia

**Keywords:** postural control, proprioception, motor imagery, virtual reality, electromyography, sensory reweighting, sensorimotor integration, cognitive style

## Abstract

Understanding the central nervous system responses to different threats of balance loss is instrumental for developments in behavioral neurophysiology and rehabilitation. Motor imagery (MI) and virtual reality (VR) are used to model postural threats. Cognitive–perceptual style, specifically field independence and field dependence, is known to modulate the postural response. However, data comparing responses to MI (internally generated threat) and VR (externally induced multisensory conflict) while accounting for cognitive–perceptual style remain insufficient. The degree of visual dependence, assessed using the Rod-and-Frame Test (RFT), was evaluated in 55 healthy young adults. Stabilographic parameters and surface electromyography were recorded during four stances (open eyes, closed eyes, MI of free fall, and VR simulation of free fall). At the systemic level, VR induced the most pronounced destabilization (*q* < 0.001). Greater RFT error (indicative of field dependence) was associated with the greatest instability (*q* < 0.01). At the neuromuscular level, VR was accompanied by increased gastrocnemius medialis activity (*q* < 0.0001). Micro-level analysis revealed significant changes in EMG signal complexity during VR and in association with RFT (*q* < 0.05 and *q* < 0.0001), suggesting differences in neuromuscular signal complexity. MI and VR differentially modulated postural control, with VR inducing stronger effects. Adaptations were detectable at multiple levels of adaptation, ranging from systemic destabilization to changes in neuromuscular coordination and micro-level muscle activity. Visual dependence was associated with greater instability. These data point to the potential value of accounting for individual variations in visual information use and highlight the need to further investigate these effects in clinical populations.

## 1. Introduction

Under perceived threat, postural control undergoes significant reorganization, shifting from automatic subcortical mechanisms toward more flexible, cognitively mediated regulation (F. B. Horak, 2006; Adkin & Carpenter, 2018). Understanding how the central nervous system differentiates and responds to different types of balance threats is instrumental for both fundamental neurophysiology and the development of effective rehabilitation technologies. Motor imagery (MI) and virtual reality (VR) are commonly used to model and study postural threats. MI and VR represent two different approaches to modeling postural threat: one aimed primarily at an internally generated cognitive challenge, the other at immersive multisensory stimulation designed to induce conflict externally. However, this dichotomy is not absolute: MI may also engage affective and perceptual processes beyond pure cognitive representation (Fujikawa et al., 2023), while VR is known to simultaneously evoke emotional, attentional, and threat-related responses alongside sensory conflict (Raffegeau et al., 2020; Baltin et al., 2026). Specifically, MI of falling activates neural networks that partially overlap with those of actual movement, inducing subtle changes in postural stability even without physical displacement (Lemos et al., 2014; Sakai et al., 2021). In contrast, exposure to a VR simulation of a fall provokes a more intense sensory conflict and emotional response, leading to significant postural alterations (Bzdúšková et al., 2022; L. M. Bikchentaeva et al., 2024; Ko et al., 2026). Both approaches allow us to examine how the central nervous system responds to threats of different origins, and both are used in neurorehabilitation to improve postural control and reduce fear of falling (Cortés-Pérez et al., 2023; Choy et al., 2023). Nevertheless, the fundamental mechanisms underlying their effects remain insufficiently differentiated.

An important individual factor shown to modulate the response to balance threat is cognitive–perceptual style, specifically field dependence and field independence (Witkin & Goodenough, 1982; Shamantseva et al., 2024). Field dependence is associated with greater reliance on visual cues and increased sensitivity to visual immersion and sensory conflicts, whereas field independence is associated with more effective use of proprioceptive and vestibular information (Maneuvrier et al., 2021; Baltin et al., 2025). Recent studies also support that individual characteristics of spatial perception significantly influence postural stability under various neurophysiological conditions (Lee et al., 2021; L. Bikchentaeva et al., 2024; Shamantseva et al., 2026).

Despite the use of MI and VR in experimental and clinical research (Boroomand-Tehrani et al., 2020; Kim & Cho, 2022), studies comparing the postural and neuromuscular responses to internal cognitive threat and external sensory conflict while accounting for individual differences in cognitive–perceptual style remain scarce. Related studies have examined MI and VR in specific contexts, including walking balance performance (Boroomand-Tehrani et al., 2020), balance training in older adults (Kim & Cho, 2022), and post-stroke rehabilitation (Choy et al., 2023). However, it remains insufficiently understood how threat type and cognitive–perceptual style jointly affect postural control across different levels of postural organization.

The aim of the present study was to determine how the type of postural threat models (MI and VR) and individual cognitive–perceptual style (field dependence/independence) modulate postural control across three levels of organization: systemic stability, neuromuscular coordination, and the temporal structure of individual muscle activity.

We hypothesized that (1) VR-induced multisensory conflict would produce deeper reorganization of postural control than MI-induced cognitive threat at different levels of organization, as VR also creates a mismatch between visual, vestibular, and proprioceptive information, whereas MI acts primarily through top-down cognitive and affective pathways without direct afferent mismatch. (2) Cognitive–perceptual style would moderate this response asymmetrically: field dependence, consistent with greater reliance on visual cues, would be associated with heightened vulnerability specifically to VR, whereas field independence would be associated with greater stability through effective reweighting toward proprioceptive and vestibular inputs. This effect would be most pronounced during VR and minimal during the eyes-closed stance, where visual input is absent for both groups.

## 2. Materials and Methods

### 2.1. Study Participants and Ethical Approval

In the present study, healthy young adults were selected as a model population to explore fundamental mechanisms of threat-induced postural reorganization, as this approach minimizes confounds associated with age-related sensory decline or neurological impairment.

Participants were recruited in the period from 26 February 2026 to 29 April 2026. Fifty-five individuals with no neurological disorders participated in the study. All reported feeling well prior to the experiment. All participants were informed of the experimental protocol and the purpose of the study and signed voluntary informed consent. Participants were able to withdraw from the study at any time. The study was conducted in accordance with the Declaration of Helsinki and was approved by the local Bioethics Committee of the Sirius Science and Technology University at its meeting on 11 June 2025. Participant data were collected and processed in anonymized form.

Exclusion criteria included neurological or neuromuscular disorders, vestibular dysfunctions, musculoskeletal conditions affecting balance, inability to perform motor imagery, and inability or unwillingness to follow the experimental protocol.

### 2.2. Stabilometry

To assess postural stability during the experimental trials, an automated Stabilan-01-2 system (JSC “OKB RITM”, Taganrog, Russia) with a sampling frequency of 50 Hz and a resolution of <0.01 mm was used. The system comprised a stabilometric platform and a recording unit (a computer running StabMed v2.13 software). The hardware component of the stabilometric system consisted of a rectangular stabilometric platform equipped with sensors on metal supports. These sensors were arranged in two independent bridge circuits for recording center-of-pressure (CoP) oscillations in the sagittal and frontal planes. The recording unit with StabMed v2.13 software processed the ground reaction force signals, computed the CoP coordinates, and derived stabilometric parameters.

For the experimental procedure, participants were asked to stand barefoot on the stabilographic platform with their feet positioned in the European stance: heels 2 cm apart, toes pointed outward, and arms resting along the trunk in a neutral position. During the trial, participants were required to maintain an upright posture and avoid sudden movements. If they experienced dizziness or any other discomfort, they were instructed to notify the researcher immediately.

The following parameters were analyzed: average linear velocity (ALV), average angular velocity (AAV), path length along frontal and sagittal planes (L(F) and L(S)), and the 60% frequency power spectrum in frontal and sagittal planes (Pw(S) and Pw(F)).

### 2.3. Virtual Reality (VR) Simulation of Free Fall

The fall simulation was presented to participants using an HTC Vive Pro 2 virtual reality headset (HTC Corporation, New Taipei City, Taiwan). The video was an interactive clip in which participants pressed a start button when ready, triggering a looping fall sequence (after each landing, the fall restarted and continued cycling for the duration of the one-minute trial).

The experimental VR application was built in Unity 6.4 (v6000.4.1f1). XR Interaction Toolkit v3.4.1 assets were used for interaction with the OpenXR HTC Vive controller profile. The following free assets were used for sounds and prefab simulation models: Nature—Essentials v1.2, Fantasy Skybox v1.6.5, Simple Sky v1.2.0, Low Poly Environment v1.0, and Rocks and Vegetation Pack URP v1.0. The corresponding Unity project and compiled simulation can be found in the data repository along with EMG data (further details are provided in the Data Availability Statement).

### 2.4. Experimental Protocol

At the beginning of the study, participants underwent body composition measurement using an InBody 770 device (Biospace Co., Ltd., Seoul, Republic of Korea), and body mass index (BMI) was calculated. Thereafter, each participant completed four trials on a stabilometric platform with different experimental tasks, all of which had been explained in detail beforehand. The rest period between trials was 2 min:

1. Open eyes (OE) trial, where participants stood with eyes open.
2. Closed eyes (CE) trial, which served as the primary control for the subsequent motor imagery (MI) trial. The CE condition was selected as the control for MI because both involve the absence of visual input, thereby isolating the additional contribution of active cognitive imagery and perceived threat.
3. Motor imagery (MI) trial, where participants were required to mentally imagine a space with a cliff, take an imaginary step down with the right foot, and then immediately transition into a state of free fall, focusing on the sensation of weightlessness or endless falling. If a landing scenario occurred prematurely, the sequence had to be repeated from the beginning. Before the trial, participants were given time to imagine this scenario and were asked to confirm that they were able to do so.
4. Virtual reality (VR) trial, where a VR simulation of free fall was presented using a VR headset.

Each trial lasted one minute (separated by 1-min rest intervals), during which stabilometry and surface electromyography were recorded. Immediately after the recording, participants were asked to rate the quality of MI using two questions on a numerical scale from 0 to 10: (1) How vividly and realistically were you able to imagine the falling process? (0—could not imagine at all; 10—extremely vivid and realistic, as in reality). (2) How strongly did you feel the threat of losing balance during imagery? (0—did not feel any threat; 10—very strongly felt a threat). If the vividness rating was below 4, that trial was excluded from further analysis.

After these stabilometry trials, participants completed the Rod-and-Frame Test (RFT) to determine their degree of visual dependence. More details on the RFT procedure are given below.

### 2.5. Assessment of Cognitive–Perceptual Style

#### Rod-and-Frame Test

The degree of visual dependence was assessed using the Rod-and-Frame Test (RFT). The RFT evaluates an individual’s perception of verticality by measuring how a tilted visual frame, presented simultaneously with a rod, affects the ability to align the rod to the true vertical. The RFT was administered using a VR headset and open-source software presented in (Josupeit, 2024). In VR, a square frame was presented against a black background, with two luminous dots inside it. Participants were required to mentally connect these dots into a line and use the VR controller to rotate it around its center so that it became strictly vertical (i.e., perpendicular to the ground; gravitational vertical, not the virtual space). The frame was presented at different tilt angles: ±18° and ±9° relative to the vertical. The initial orientation of the line defined by the dots also varied, at ±20° and ±10°. Each participant completed 10 trials (excluding one tutorial trial) presented with different combinations of frame angles and line angles; the presentation order was randomized. When the participant felt that the line was positioned exactly perpendicular to the ground, they pressed the lower button on the VR controller, after which the next configuration of the frame and dots appeared. Based on the collected data, the median absolute error (deviation from true vertical) was calculated in degrees. A higher median RFT error suggests a greater degree of visual dependence.

### 2.6. Electromyography Recording

During each stabilometry trial, electromyography (EMG) was recorded from the bellies of the following muscles of the dominant leg: gastrocnemius medialis (GM), soleus (Sol), gastrocnemius lateralis (GL), and tibialis anterior (TA). Single-channel Avanti sensors (Delsys Inc., Natick, MA, USA) and the Delsys Trigno Discover software v1.6.4.0 were used at a sampling frequency of 2148 Hz. Sensor placement on the target muscles was performed in accordance with the SENIAM project recommendations and the surface EMG atlas (Hermens et al., 2000; Barbero et al., 2012).

### 2.7. Data Analysis

Data analysis was performed using Python v3.12.2. Surface electromyographic (sEMG) data were band-pass filtered at 30–400 Hz, and a 50 Hz notch filter was applied using SciPy v1.17.1 (Virtanen et al., 2020) prior to analysis. All EMG records were truncated to a uniform length of 60 s. The EMG envelopes were derived from rectified EMG signals smoothed using a 1000 ms moving average. A 1000 ms window was selected as a compromise between noise reduction and the preservation of slow postural modulation frequencies (<1 Hz) to obtain a smooth EMG envelope suitable for computing co-contraction indices and area-under-curve measures over the 60-s trials.

To quantify overall muscle effort, the area under the EMG envelope (iEMG) was calculated using Simpson’s rule approximation as implemented in SciPy. This area served as a relative measure of overall muscle recruitment, with larger values indicating greater muscle effort. For comparison, iEMG values were normalized per participant relative to their individual baseline reference stance (OE) to quantify relative changes in muscle effort.

The stiffening strategy was assessed using EMG-signal analysis of agonist–antagonist muscle co-contraction. Muscle co-contraction was quantified from EMG envelopes using the Falconer–Winter co-contraction index (CCI_f_) as described in (Falconer & Winter, 1985; Falk et al., 2022). CCI_f_ is derived from the ratio of two muscles’ signals and reaches its maximum when the EMG signals of both muscles are equal (Equation (1)).

CCI_f_ = (2 × Low)/(Low + High), (1)

where “Low” represents the muscle with the lowest EMG value for that time period.

The temporal organization of surface EMG activity may vary with neuromuscular state and task demands. To gain insight into the neuromuscular state, EMG signal complexity was quantified using approximate entropy (ApEn) (Rampichini et al., 2020). ApEn was computed on the raw (unsmoothed, band-pass filtered) EMG signal, rather than on the smoothed envelope, to preserve the temporal complexity of the signal. ApEn was computed as described in (Pincus, 1991; Rampichini et al., 2020; Flood & Grimm, 2021) using EntropyHub v2.0 (Flood & Grimm, 2021) from raw EMG signals for all 1-s windows of the signal, with embedding size (*m*) of 2 and filtering threshold (*r*) of 0.2 standard deviations of a given data subset (default parameters). Overall signal entropy was assessed as the median. Lower ApEn values reflect higher signal regularity (i.e., reduced complexity). ApEn calculation comprises the following steps:

1. For a given sequence of length *N*, define a series of vectors *X*(*i*) of length *m*, consisting of consecutive data points starting from index *i* to *N* − *m* + 1.
2. Define the distance between two vectors *X*(*i*) and *X*(*j*) as the maximum of the absolute differences between their respective components.
3. For each *i*, count the number of vectors *X*(*j*) that fall within the distance *r* from *X*(*i*). Calculate the relative fraction of matched vectors, denoted $C_{i}^{m}$(*r*). Note that *j* takes on all values from 1 to *N* − *m* + 1.
4. Calculate the average of the sum of natural logarithms of these fractions: ϕ^m^(*r*) = Σ ln($C_{i}^{m}$(*r*))/(*N* − *m* + 1).
5. Calculate ApEn as ApEn(*m*, *r*, *N*) = ϕ^m^(*r*) − ϕ^m+1^(*r*).

### 2.8. Statistical Analysis

Statistical comparisons were carried out using NumPy v1.26.4 (Harris et al., 2020), SciPy v1.17.1 (Virtanen et al., 2020), and statsmodels v0.14.6 (Seabold & Perktold, 2010).

RFT error and all compared outcome variables were standard-scaled before the analyses. Three omnibus tests were assessed: the effect of stance (M1a), the effect of RFT error (M1b), and the Stance × RFT error interaction (M2). First, omnibus comparisons across stances (OE, CE, MI, VR) were performed using a linear mixed model (LMM; formula: ~Stance + RFT error, reference = OE; random intercepts for subject, identity link function). A Wald test was used to compute the omnibus *p*-value for the stance effect (M1a). The RFT error model coefficient *p*-value was used for the overall omnibus assessment of the RFT error effect (M1b). Second, an omnibus test of the Stance × RFT error interaction was performed (LMM; formula: ~Stance + RFT error + Stance:RFT error, reference = OE; random intercepts for subject, identity link function), with a Wald test applied to compute the interaction omnibus *p*-value (M2). The Benjamini–Hochberg False Discovery Rate procedure (FDR-BH) was then applied to adjust for multiple comparisons across all outcome variables. M1a, M1b, and M2 were each adjusted independently. Omnibus comparisons were considered significant at an adjusted *p*-value (*q*) < 0.05. In the case of a significant omnibus test for a given outcome, post hoc comparisons were performed for that outcome by refitting the LMMs (M1 and M2) with the appropriate reference levels, and the resulting *p*-values were FDR-BH-adjusted. For stance (M1a), three contrasts were tested: OE vs. VR, CE vs. MI, and MI vs. VR. For the interaction (M2), three contrasts were tested: OE:RFT error vs. VR:RFT error, CE:RFT error vs. MI:RFT error, and MI:RFT error vs. VR:RFT error. Post hoc comparisons were considered significant at an adjusted *p*-value (*q*) < 0.05.

Data visualizations were generated using Matplotlib v3.10.8 (Hunter, 2007) and seaborn v0.13.2 (Waskom, 2021). Analyses were performed in Python v3.12.2.

Due to the relative novelty of the research question, average expected figures assuming a medium effect size were used for the power analysis. The sample size was evaluated using G*Power 3.1.97. Because G*Power does not support mixed LMM power estimation directly, a repeated-measures ANOVA (within-subject) was used as an approximation. Effect size f was set to 0.25, alpha error = 0.05, power = 0.80, number of groups = 2 (reflecting the expected visual dependence spectrum), number of measurements = 4, Corr among rep measures = 0.5, and nonsphericity correction ε = 1. The estimated required sample size was 36.

A post hoc power analysis for the mixed LMM model was additionally performed with “simr” v1.0.11 in R v4.5.3 for each hypothesis family. For significant stance comparisons, observed power was around 99% (99.60, 100.0); for the association with RFT, it ranged from 83.30% (80.84, 85.56) to 89.60% (87.54, 91.42). For the interaction, in the post hoc OE:RFT vs. VR:RFT comparison, observed power varied from 99.50% (98.84, 99.84) to 99.90% (99.44, 100.00), consistent with the observed *q*-values.

## 3. Results

### 3.1. Study Participants

Participant characteristics, including age, sex, body mass index (BMI), and results of the Rod-and-Frame Test (RFT), are presented in Table 1.

The median age was 25 years (interquartile range (IQR): 24.00; 28.00). All participants reported a sufficient level of vividness during MI, and no trials were excluded.

### 3.2. Biomechanical Level of Postural Control

**Figure 1:**
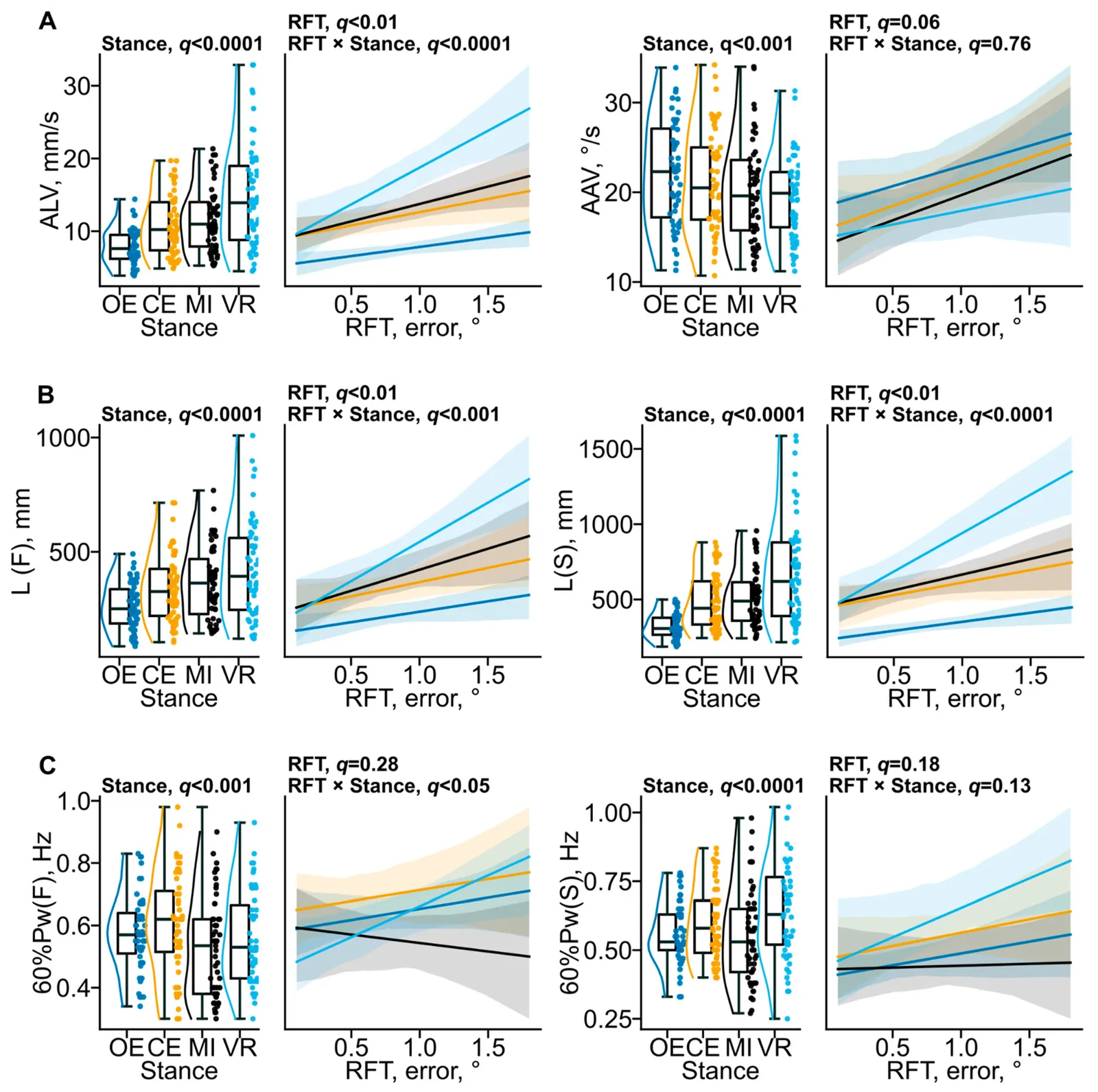
Comparison of center-of-pressure (CoP) (**A**) average linear velocity (ALV) and average angular velocity (AAV), (**B**) path length in the frontal (L(F)) and sagittal plane (L(S)), and (**C**) 60% power frequency in the frontal (60%Pw(F)) and sagittal plane (60%Pw(S)) across stances with open eyes (OE), closed eyes (CE), motor imagery (MI), and virtual reality (VR), and in association with the degree of field dependence as estimated by RFT error. LMMs: ~Stance + RFT (M1) and Stance × RFT (M2), Wald test for omnibus values. FDR-BH adjustment per omnibus family.

Stabilometric parameters serve as indicators of systemic postural stability. We first analyzed stability levels across all stances. The greatest differences were observed between the open-eyes (OE) control stance and the virtual reality (VR) condition (Figure 1A–C).

Median linear velocity (ALV) increased gradually with stances (*q* < 0.0001) (Figure 1A). It ranged from 7.62 mm/s (6.21; 9.50) in OE to 10.23 mm/s (7.37; 14.00) in closed eyes (CE) to 10.98 mm/s (7.9; 14.01) in motor imagery (MI) to 13.91 mm/s (8.8; 18.98) in VR. Significant increases were observed between OE and VR (*q* < 0.0001) and between MI and VR (*q* < 0.0001). Notably, differences between CE and MI were not significant (*q* = 0.17). Contrary to ALV, average angular velocity (AAV) demonstrated a significant decreasing trend (*q* < 0.001). It ranged from 22.3°/s (17.2; 27.1) in OE to 20.5°/s (16.95; 25.0) in CE to 19.6°/s (15.75; 23.6) in MI to 19.9°/s (16.1; 22.25) in VR. Significant differences were observed between OE and VR (*q* < 0.0001), whereas CE and MI (*q* = 0.11) and MI and VR (*q* = 0.58) did not differ significantly.

When comparing center-of-pressure (CoP) path length (Figure 1B) in the frontal (L(F)) and sagittal plane (L(S)), a similar increasing pattern to ALV was observed. The largest difference was observed between OE and VR conditions (*q* < 0.0001). For L(F), the CoP path length was 250.6 mm (186.3; 336.45) in OE and reached 392.8 mm (246.0; 560.85) in VR. For L(S), it was 307.1 mm (265.5; 378.35) and 619.8 mm (390.5; 878.75), respectively.

Power frequency analysis (Figure 1C) revealed a specific exception to the OE-VR divergence pattern in the frontal plane: 60% power frequency in the frontal plane (60%Pw(F)) did not differ significantly between OE and VR settings (*q* = 0.92), indicating maintenance of a similar median frequency concentrating in the lower mid-frequency range even under increased visual load. Overall 60%Pw(F) between stances were the following: 0.57 Hz (0.51; 0.64), 0.62 Hz (0.52; 0.71), 0.54 Hz (0.38; 0.62), and 0.53 Hz (0.43; 0.66) in OE, CE, MI, and VR, respectively. Notably, the largest significant difference was observed between CE and MI stances (*q* < 0.001). VR did not differ significantly from MI (*q* = 0.07). At the same time, when comparing 60% power frequency in the sagittal plane (60%Pw(S)), VR again demonstrated a significant shift from the OE condition (*q* < 0.0001), marking a substantial increase. 60%Pw(S) between stances were the following: 0.53 Hz (0.5; 0.63), 0.58 Hz (0.49; 0.68), 0.53 Hz (0.42; 0.65), and 0.63 Hz (0.52; 0.76) in OE, CE, MI, and VR, respectively. Additionally, it was observed that CE differed significantly from MI (*q* < 0.05) and VR from MI (*q* < 0.0001).

Next, we assessed the association between stabilometric parameters and the degree of field dependence as estimated by RFT error. Significant associations (Stance + RFT) were found for ALV (*q* < 0.01), L(F) (*q* < 0.01), and L(S) (*q* < 0.01). When comparing the rate of postural parameter change across the stances (Stance × RFT), it was observed that the increase in ALV was greater in VR than in OE (*q* < 0.0001), that the rate of change did not differ between CE and MI (*q* = 0.53), and that the rate differed between MI and VR (*q* < 0.001). Similarly, for L(F) and L(S), a difference in rate was observed between OE and VR (*q* < 0.0001 for both parameters) and between MI and VR (*q* < 0.01 and *q* < 0.001, respectively) but not between CE and MI (*q* = 0.39 and *q* = 0.66, respectively).

Thus, at the systemic level of postural control, cognitive–visual load, particularly in the VR paradigm, was associated with more pronounced destabilization. The association between stabilometric parameters and the degree of field dependence suggests that individual differences in visuospatial information processing may explain part of the destabilization effect, especially in VR, where a higher degree of visual dependence was associated with more pronounced instability.

Although postural responses differed across conditions, these differences cannot be attributed unequivocally to a single factor given the fixed testing order. Nonetheless, the sharp VR-related destabilization and its association with field dependence (as assessed by RFT error) are difficult to explain by fatigue or habituation alone. Additionally, in a subset of 5 participants, we performed an exploratory analysis to evaluate whether a neutral VR condition exhibited effects similar to those of free-fall VR or OE. In this subset, the testing order was OE → CE → MI → VR → neutral VR. In the neutral VR condition, the outer camera was turned on so that the participant could see the room, as in the OE condition. These analyses demonstrated that the neutral VR condition did not differ substantially from the OE condition for the majority of parameters (OE vs. neutral VR; all *p*-values unadjusted: ALV, *p* = 0.83; AAV, *p* = 0.41; L(F), *p* = 0.54; L(S), *p* = 0.96; 60%Pw(S), *p* = 0.74), with the exception of 60%Pw(F), which differed significantly (*p* < 0.001). This suggests that the observed effects were primarily driven by exposure to the VR free-fall simulation, rather than by the VR headset or fatigue. This observation is consistent with previous findings (Mihalik et al., 2008; Pujiartati et al., 2020), which reported no significant association between VR headset exposure and changes in stabilometric measures, as well as no substantial fatigue during stabilometric trials under 30 min. These exploratory analyses can be found in Supplementary File S1.

### 3.3. Neuromuscular Level of Postural Control

**Figure 2:**
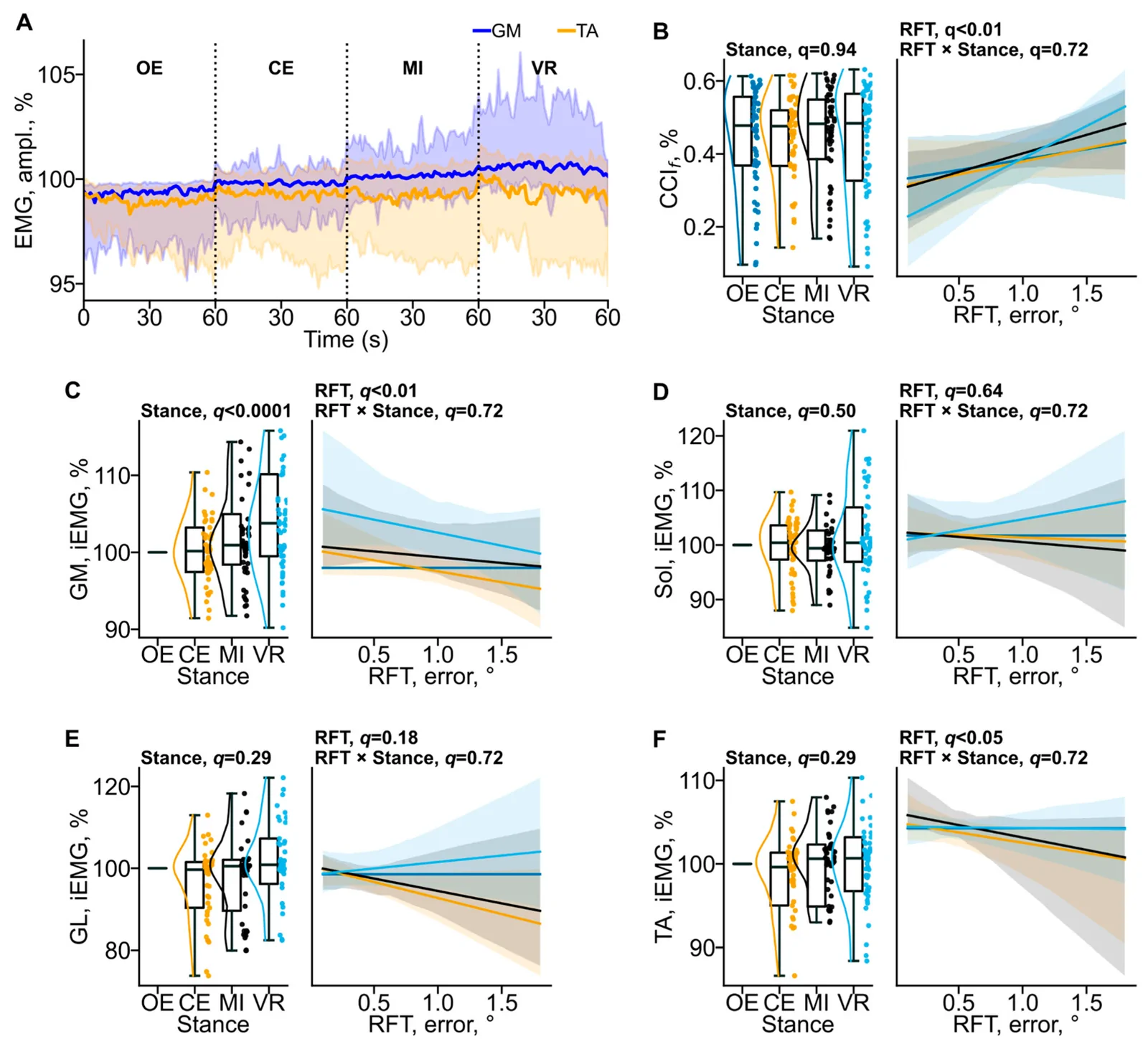
Electromyographic activity of the gastrocnemius medialis (GM), soleus (Sol), gastrocnemius lateralis (GL), and tibialis anterior (TA) muscles in stances with open eyes (OE), closed eyes (CE), motor imagery (MI), and virtual reality (VR), and in association with the degree of field dependence as estimated by RFT error. (**A**) Median muscle activity (solid line) with interquartile range (shaded area) of the TA and GM normalized per participant relative to their individual baseline reference stance (OE) median level. (**B**) Co-contraction index (CCI_f_) (TA, GM). (**C**–**F**) Area under the EMG envelope for all muscles normalized per participant relative to their individual baseline reference stance (OE) level. LMMs: ~Stance + RFT (M1) and Stance × RFT (M2), Wald test for omnibus values. FDR-BH adjustment per omnibus family.

Analysis of electromyographic (EMG) activity of the muscles suggested condition-dependent modulation of neuromotor control (Figure 2). Figure 2A presents the median curves of the smoothed EMG envelope (normalized per participant relative to their individual baseline reference stance (OE) to quantify relative changes in muscle effort) with IQR for the gastrocnemius medialis (GM) and tibialis anterior (TA) muscles across all participants, showing the behavior of the EMG signal across all stances.

Further analysis of co-contraction of TA and GM (Figure 2B) across all stances demonstrated no significant variation (*q* = 0.94). The median CCI_f_ value was 0.48 (0.36; 0.55). Association analysis demonstrated an association between RFT and CCI_f_, with larger CCI_f_ values associated with greater RFT error (*q* < 0.01). No interaction effects were observed (*q* = 0.72).

Subsequent analysis of overall muscle effort (Figure 2C), estimated as the area under the EMG envelope (iEMG) for each muscle individually (normalized per participant relative to their individual baseline reference stance (OE) to quantify relative changes in muscle effort), revealed a significant increase in activity of the GM muscle from OE to VR (*q* < 0.0001). Specifically, the increase for GM was 3.78% (−0.51; 10.15) (*q* < 0.0001). The soleus (Sol), gastrocnemius lateralis (GL), and tibialis anterior (TA) muscle effort did not demonstrate significant change across stances (*q* = 0.50, *q* = 0.29, and *q* = 0.29, respectively). Association analysis demonstrated an association between RFT and GM and TA effort %, with smaller effort % values associated with greater RFT error (*q* < 0.01 and *q* < 0.05, respectively). No interaction effects were observed (*q* = 0.72).

Thus, at the neuromuscular level of postural control, VR was associated with increased GM activity compared to the control condition (OE). This reorganization suggests greater plantarflexor engagement. The association between muscle engagement and the degree of field dependence, as estimated by RFT error, suggests that neuromuscular reorganization may differ depending on individual differences in visuospatial information processing.

### 3.4. Micro-Level Organization of Muscle Activity

**Figure 3:**
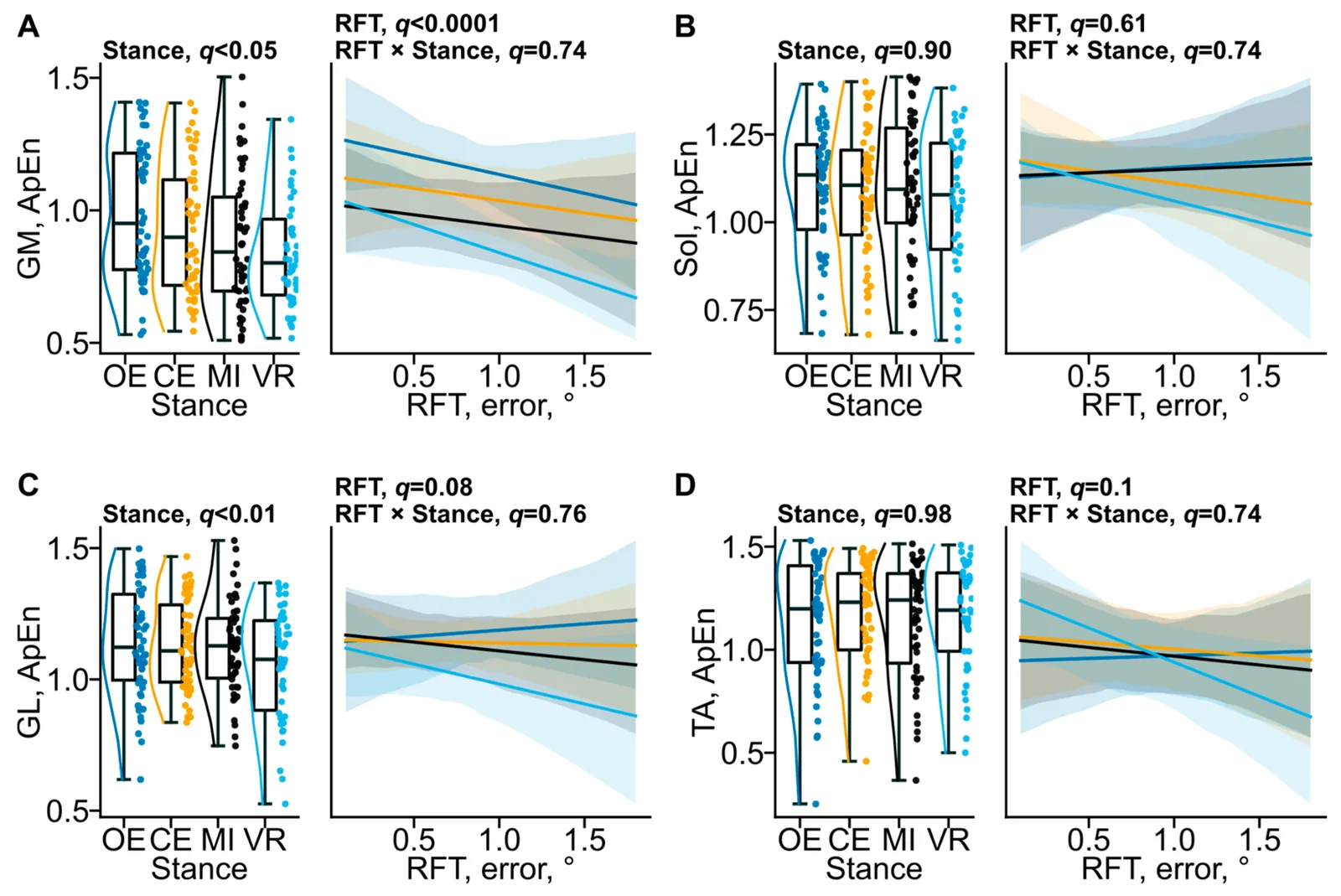
Comparison of electromyographic signal complexity measured by approximate entropy (ApEn) for the gastrocnemius medialis (GM), gastrocnemius lateralis (GL), tibialis anterior (TA), and soleus (Sol) muscles in stances with open eyes (OE), closed eyes (CE), motor imagery (MI), and virtual reality (VR), and in association with the degree of field dependence as estimated by RFT error: (**A**) GM; (**B**) Sol; (**C**) GL; (**D**) TA. LMMs: ~Stance + RFT (M1) and Stance × RFT (M2), Wald test for omnibus values. FDR-BH adjustment per omnibus family. ApEn parameters: embedding dimension (*m*) = 2 and tolerance (*r*) = 0.2 × SD.

To assess the complexity of the EMG signal of muscle activation, approximate entropy (ApEn) was computed. Both a significant trend of change (*q* < 0.05) across stances and a significant group effect (*q* < 0.0001) were observed. The comparison results are presented in Figure 3.

The ApEn of the EMG signals showed a relative decrease in signal complexity (*q* < 0.05), with the highest entropy observed in the OE stance and the lowest in the VR stance for GM (*q* < 0.05) and GL (*q* < 0.01). The largest effect was observed for GM, with a median decrease of 15% (from 0.95 (0.78; 1.22) to 0.80 (0.68; 0.97), *q* < 0.01). For GL, this change was 5% (from 1.12 (1.00; 1.32) to 1.08 (0.88; 1.22), *q* < 0.001). Additionally, for GL, a significant difference between MI and VR was observed (*q* < 0.001).

Association analysis demonstrated an association between RFT and GM signal complexity, with smaller signal complexity values associated with greater RFT error (*q* < 0.0001). No interaction effects were observed (*q* = 0.74).

The reduction in ApEn observed for GM and GL during VR indicates greater temporal regularity of the surface EMG signal. This pattern is compatible with a more constrained or synchronized organization of neuromuscular output (Zhou et al., 2011; Chen et al., 2018). However, the present data do not permit identification of the exact physiological source of this signal regularity, such as motor unit synchronization, changes in the active motor unit pool, or altered common synaptic input. Higher ApEn values associated with lower RFT error indicate lower temporal regularity of the surface EMG signal, which may reflect a more flexible neuromuscular output, although this interpretation cannot be confirmed with the present approach. At the same time, the specificity of the degree of visual dependence (as estimated by RFT error) association with GM may be related to its role in postural control under cognitive–visual load. Nevertheless, the present approach precludes a more precise conclusion.

We additionally recalculated the ApEn statistics using tolerance coefficients *r* ∈ {0.1, 0.15, 0.2, 0.25} and embedding dimensions *m* of both 2 and 3. The results, albeit different in amplitude, showed a similar pattern across parameter choices for both *m* = 2 and *m* = 3, with a consistent GM ApEn change effect across stances emerging from *r* > 0.15 onward; more variation was observed for associations with RFT, although the general trend was preserved (Supplementary File S2).

## 4. Discussion

The present study suggests that motor imagery (MI) of falling and virtual (VR) simulation of falling differentially modulate postural control in healthy adults, with a greater magnitude of effect for VR. A notable observation is that differences are detectable at multiple levels of adaptation, ranging from systemic destabilization in the sagittal plane to specific changes in neuromuscular coordination and micro-level patterns of muscle activity. Additionally, the present analysis offers evidence of the effects of cognitive–perceptual style (as assessed by median error in the Rod-and-Frame Test (RFT)) on this organization: greater RFT error (indicative of field dependence) was associated with higher instability in VR simulation, consistent with the hypothesis that cognitive–perceptual style plays a role in filtering afferent information. Furthermore, RFT results were associated with distinct electromyographic signal complexity.

At the systemic biomechanical level, our data showed that the VR paradigm induces a more pronounced postural response than MI, characterized by increases in linear velocity and path length of center-of-pressure oscillations. This is consistent with previous studies (Raffegeau et al., 2020; Dewan et al., 2023), indicating that visual–vestibular conflict induced by VR disrupts the integration of sensory inputs, forcing the central nervous system to reweight proprioceptive signals. Notably, the MI condition induced less pronounced changes in most stabilometric parameters relative to the closed-eyes stance and the VR condition. This suggests that an endogenous threat, lacking direct afferent conflict, may engage postural control networks to a lesser degree than the multimodal stimulation provided by VR. In particular, MI likely also involves affective and anticipatory processes that partially overlap with those engaged during VR exposure, without triggering full protective balance mechanisms.

This study’s data additionally provide evidence for a modulatory role of cognitive–perceptual style. Greater RFT error was associated with more pronounced destabilization specifically in the VR condition, consistent with a greater reliance on visual cues for spatial orientation. While sensory conflict is likely the dominant driver of the VR-induced response, it is important to note that the VR condition also involved emotional arousal, perceived threat, and increased attentional demands. The present design does not allow dissociation of these components, and the observed effects should be interpreted as reflecting the composite VR experience rather than only sensory conflict. In contrast, lower RFT error (indicative of field independence) was associated with better compensation for the effects of VR stimulation, suggesting a greater capacity to ignore the VR experience and rely on somatosensory information. This observation suggests that individual perceptual characteristics warrant consideration in the development of VR protocols for rehabilitation.

It is known that postural threat is accompanied by increased co-activation of ankle antagonists, which increases ankle joint stiffness to stabilize posture (Nashner, 1982; F. Horak et al., 1989; Loram et al., 2001; Carpenter et al., 2001). In the present study, VR fall simulation induced a threat of postural instability, but no change in muscle co-activation was observed. At the same time, the increased activity of the gastrocnemius was detected. It may reflect an attempt to compensate for the subjectively perceived forward body displacement induced by the VR environment (Li et al., 2025; Hansson et al., 2026). Such changes are considered a manifestation of dynamic sensory reweighting, in which the central nervous system redistributes the contribution of different sensory channels and alters the organization of the motor response under conditions of afferent conflict.

Analysis of the complexity of muscle electromyographic (EMG) signals using approximate entropy offered further insight into the organization of neuromuscular output under different postural-threat conditions and with respect to cognitive–perceptual style. VR stimulation was associated with a significant decrease in EMG signal entropy in the gastrocnemius medialis, reflecting a reduction in signal complexity. This observation is consistent with the finding that entropy measures are sensitive to changes in muscle activation patterns (Xie et al., 2010; Sun et al., 2014; Stins et al., 2015). Concurrently, lower RFT error was associated with overall higher EMG complexity across stances, suggesting a difference in the temporal organization of neuromuscular activity signals. The lower EMG complexity suggests a more regular and less variable pattern of muscle activity. This observation is consistent with the evidence that EMG entropy measures are sensitive to changes in the organization and adaptability of neuromuscular activity (Kędziorek & Błażkiewicz, 2020; Wu, 2024). Furthermore, the transition from open eyes to VR was accompanied by a decrease in entropy in the posterior leg muscles, specifically in the ankle agonist gastrocnemius medialis, whereas the entropy of the tibialis anterior did not change significantly. This muscle-specific reduction in entropy may reflect increased temporal regularity and a more constrained organization of agonist muscle activity during VR. Such a pattern aligns with the view that increased sensory and cognitive demands can reduce the flexibility of neuromuscular control and promote more stable, predictable activation patterns (Brock et al., 2023; Veronez et al., 2024), although this interpretation cannot be confirmed with the present approach. Taken together, the observed approximate entropy differences indicate that both the type of threat-of-falling paradigms and degree of visual dependence (as estimated by RFT) are associated with the temporal organization of neuromuscular activity.

The present study has several limitations. First, the validation of the MI condition relied exclusively on subjective self-report questionnaires. Second, the protocol did not include subjective or objective measurements of cybersickness or visual disorientation. Third, the biomechanical and neuromotor analysis was restricted to the ankle joint muscles of the leading leg. While this provides valuable data on local ankle strategies, it offers a limited view of whole-body postural reorganization. Fourth, the study sample consisted of a relatively small number of healthy young adults. Consequently, the findings cannot be directly generalized to clinical populations or older adults. Fifth, the conditions were administered in a fixed order. This order was chosen deliberately—MI before VR to prevent carryover of visual reference, and VR last to prevent any potential lasting effect of VR exposure on subsequent conditions. However, this design choice means that the condition is confounded with trial order, and we cannot fully separate the effects of the VR condition from those of order. Nonetheless, several considerations suggest that order effects were likely to be limited in magnitude. In particular, each trial lasted only one minute, with one minute of rest between the trials, for a total session duration of approximately 7–8 min—leaving no substantial time for fatigue or habituation to develop. Accordingly, accumulated evidence suggests that stabilometric experiments lasting under 30 min (Pujiartati et al., 2020) are unlikely to induce significant fatigue. Moreover, habituation or fatigue would predict a gradual change in sway across conditions, which does not match the sharp, VR-specific change observed. It cannot be completely ruled out that some of the postural destabilization observed in VR can be attributable to VR headset exposure, although current research, together with our exploratory neutral VR analysis (Supplementary File S1), suggests these effects are insignificant (Mihalik et al., 2008; Pujiartati et al., 2020). Nonetheless, it should be noted that the present study design did not permit a direct assessment of order effects, and they were not systematically tested. Finally, although a neutral VR condition was included in an exploratory subset of participants (Supplementary File S1), its limited administration across the full sample represents a notable limitation, warranting further analysis aimed at disentangling the effects of immersion, visual stimulation, emotional arousal, attentional demand, perceived threat, VR headset exposure, cybersickness, and visual disorientation. It should be noted that participants’ sex was not included in the regression analyses. This was done because the primary research question of the present analysis concerns the association between RFT and postural parameters rather than any potential demographic determinants of RFT. As a sensitivity check, we reran the analyses while including sex as a covariate. The pattern of results was unchanged (shifts in *q*-value thresholds, with no substantial changes), supporting the robustness of the observations. The main analyses are reported without demographic covariates, and the adjusted results can be found in Supplementary File S3.

## 5. Conclusions

The present study suggests that the postural response to motor imagery of falling and virtual simulation of falling is detectable at multiple levels of adaptation, ranging from the systemic level to neuromuscular coordination and to the micro-level organization of muscle activity. Degree of visual dependence (as estimated by the Rod-and-Frame Test (RFT)) appears to modulate these changes: greater RFT error (indicative of field dependence) was associated with increased vulnerability to simulation in virtual reality, whereas lower RFT error was associated with greater stability. Furthermore, RFT error was associated with distinct electromyographic signal complexity. These data point to the potential value of accounting for individual variations in visual information use. These results further suggest that graded virtual reality exposure and multisensory support warrant investigation and highlight the need to examine these effects in clinical populations.

## Figures and Tables

**Table 1:** Demographics and functional characteristics of the study cohort. Data are presented as median and IQR (interquartile range) or *n* (%).

Variable	Participants *n* = 55
Age, yrs	25.00 (24.00; 28.00)
Sex, male	27 (49.09%)
BMI, kg/m^2^	21.60 (19.70; 24.45)
Median RFT, error, °	0.80 (0.42; 1.08)

RFT = Rod-and-Frame Test; BMI = body mass index.

## Data Availability

The original data presented in the study are openly available in the Zenodo repository at https://doi.org/10.5281/zenodo.22643845 (accessed on 2 October 2026).

## Supplementary Materials

The following supporting information can be downloaded at https://www.mdpi.com/article/10.3390/bs16101907/s1, Supplementary File S1: Exploratory analysis of VR headset exposure. Supplementary File S2: Analysis of the effect of parameter selection on ApEn results. Supplementary File S3: Regression analysis results with sex included as a covariate.

## Abbreviations

The following abbreviations are used in this manuscript:

ApEn	Approximate entropy
CCI	Co-contraction index
CoP	Center of pressure
CE	Closed eyes
EMG	Electromyography
MI	Motor imagery
OE	Open eyes
VR	Virtual reality
RFT	Rod-and-Frame Test
