# Supplementary material for: The Effects of Cognitive–Perceptual Style on the Multilevel Organization of Postural Control During Motor Imagery and Immersive Virtual Reality
Source: Behav Sci (Basel). 2026 Oct 15;16(10):1907. doi: 10.3390/bs16101907 (PMC13701233; doi:10.3390/bs16101907)
Supplement: Supplementary file 1 [file behavsci-16-01907-s001.zip › Supplementary File S1/Supplementary File S1.1.pdf]

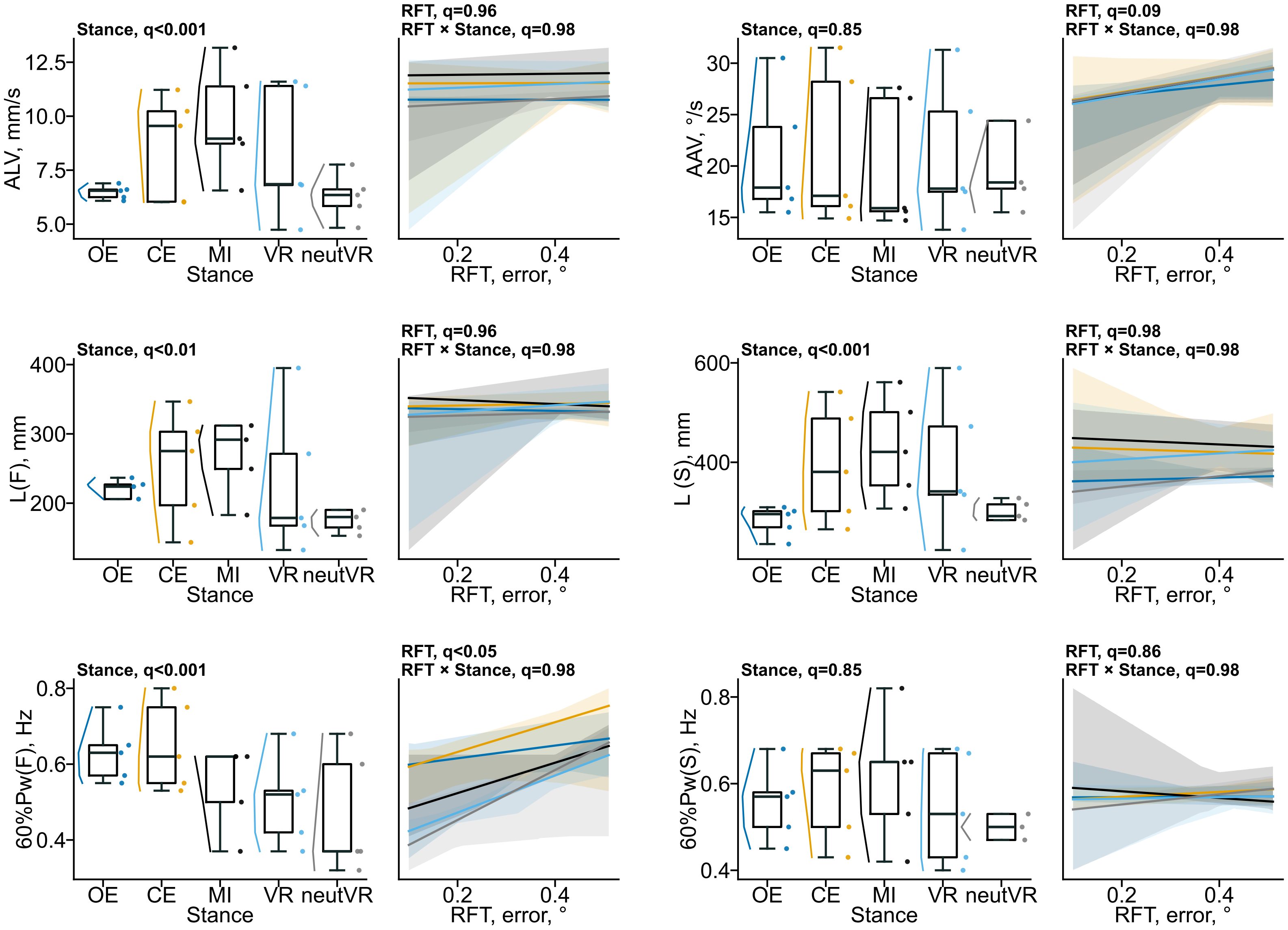

Stance, q<0.001
ALV, mm/s
12.5
10.0
7.5
5.0
OE CE MI VR neutVR
Stance
RFT, q=0.96
RFT × Stance, q=0.98
0.2 0.4
RFT, error, °
Stance, q=0.85
AAV, °/s
30
25
20
15
OE CE MI VR neutVR
Stance
RFT, q=0.09
RFT × Stance, q=0.98
0.2 0.4
RFT, error, °
Stance, q<0.01
L(F), mm
400
300
200
OE CE MI VR neutVR
Stance
RFT, q=0.96
RFT × Stance, q=0.98
0.2 0.4
RFT, error, °
Stance, q<0.001
L(S), mm
600
400
OE CE MI VR neutVR
Stance
RFT, q=0.98
RFT × Stance, q=0.98
0.2 0.4
RFT, error, °
Stance, q<0.001
60%Pw(F), Hz
0.8
0.6
0.4
OE CE MI VR neutVR
Stance
RFT, q<0.05
RFT × Stance, q=0.98
0.2 0.4
RFT, error, °
Stance, q=0.85
60%Pw(S), Hz
0.8
0.6
0.4
OE CE MI VR neutVR
Stance
RFT, q=0.86
RFT × Stance, q=0.98
0.2 0.4
RFT, error, °
