# Supplementary material for: The Effects of Cognitive–Perceptual Style on the Multilevel Organization of Postural Control During Motor Imagery and Immersive Virtual Reality
Source: Behav Sci (Basel). 2026 Oct 15;16(10):1907. doi: 10.3390/bs16101907 (PMC13701233; doi:10.3390/bs16101907)
Supplement: Supplementary file 1 [file behavsci-16-01907-s001.zip › Supplementary File S2/Supplementary File S2.1 (m=2, r=0.1).pdf]

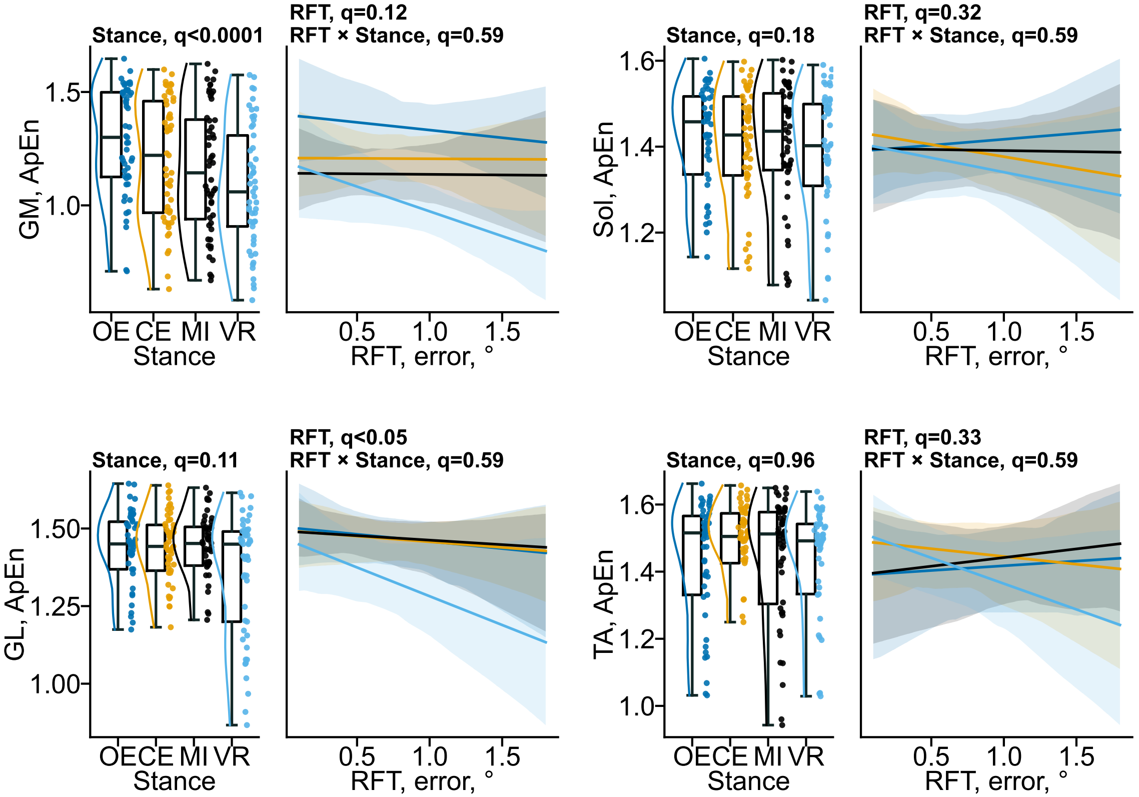
Stance, q<0.0001
RFT, q=0.12
RFT × Stance, q=0.59
GM, ApEn
1.5
1.0
OE CE MI VR
Stance
0.5 1.0 1.5
RFT, error, °
Stance, q=0.18
RFT, q=0.32
RFT × Stance, q=0.59
Sol, ApEn
1.6
1.4
1.2
OE CE MI VR
Stance
0.5 1.0 1.5
RFT, error, °
Stance, q=0.11
RFT, q<0.05
RFT × Stance, q=0.59
GL, ApEn
1.50
1.25
1.00
OE CE MI VR
Stance
0.5 1.0 1.5
RFT, error, °
Stance, q=0.96
RFT, q=0.33
RFT × Stance, q=0.59
TA, ApEn
1.6
1.4
1.2
1.0
OE CE MI VR
Stance
0.5 1.0 1.5
RFT, error, °
