# Supplementary material for: The Effects of Cognitive–Perceptual Style on the Multilevel Organization of Postural Control During Motor Imagery and Immersive Virtual Reality
Source: Behav Sci (Basel). 2026 Oct 15;16(10):1907. doi: 10.3390/bs16101907 (PMC13701233; doi:10.3390/bs16101907)
Supplement: Supplementary file 1 [file behavsci-16-01907-s001.zip › Supplementary File S2/Supplementary File S2.2 (m=2, r=0.15).pdf]

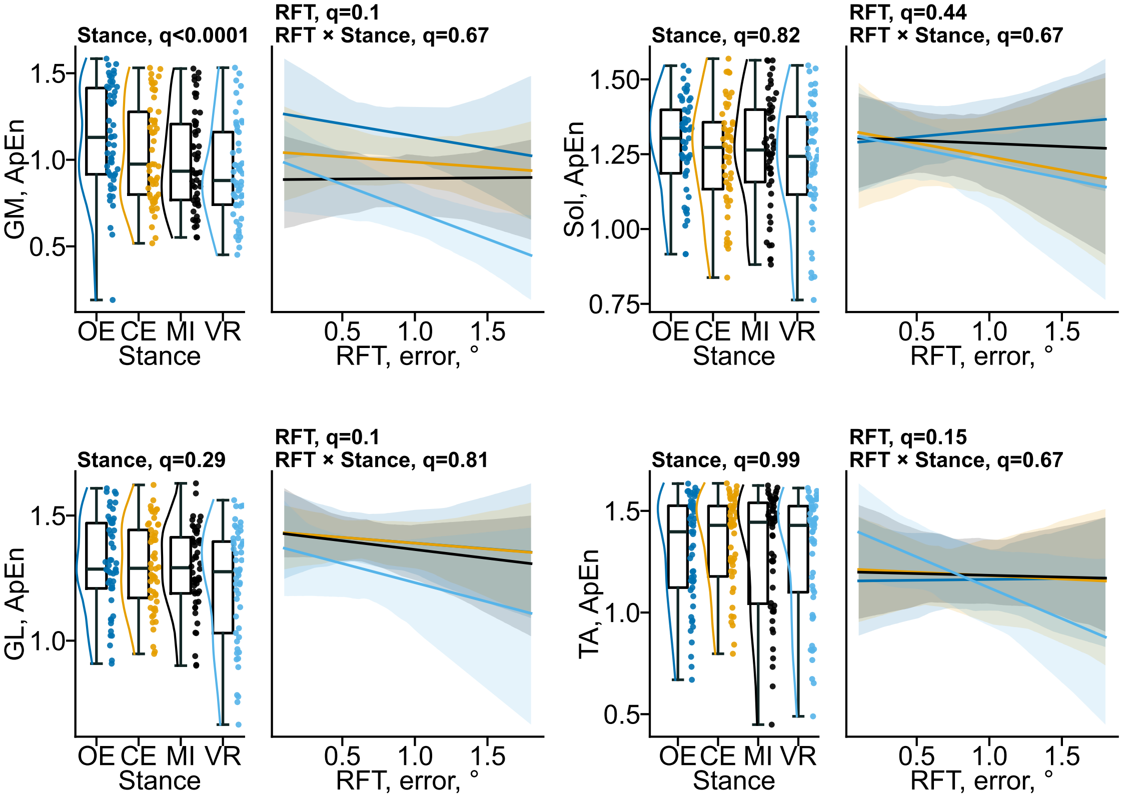

Stance, q<0.0001
RFT, q=0.1
RFT × Stance, q=0.67
GM, ApEn
Stance
RFT, error, °
Stance, q=0.82
RFT, q=0.44
RFT × Stance, q=0.67
Sol, ApEn
Stance
RFT, error, °
Stance, q=0.29
RFT, q=0.1
RFT × Stance, q=0.81
GL, ApEn
Stance
RFT, error, °
Stance, q=0.99
RFT, q=0.15
RFT × Stance, q=0.67
TA, ApEn
Stance
RFT, error, °
OE CE MI VR
