# Supplementary material for: The Effects of Cognitive–Perceptual Style on the Multilevel Organization of Postural Control During Motor Imagery and Immersive Virtual Reality
Source: Behav Sci (Basel). 2026 Oct 15;16(10):1907. doi: 10.3390/bs16101907 (PMC13701233; doi:10.3390/bs16101907)
Supplement: Supplementary file 1 [file behavsci-16-01907-s001.zip › Supplementary File S2/Supplementary File S2.3 (m=2, r=0.25).pdf]

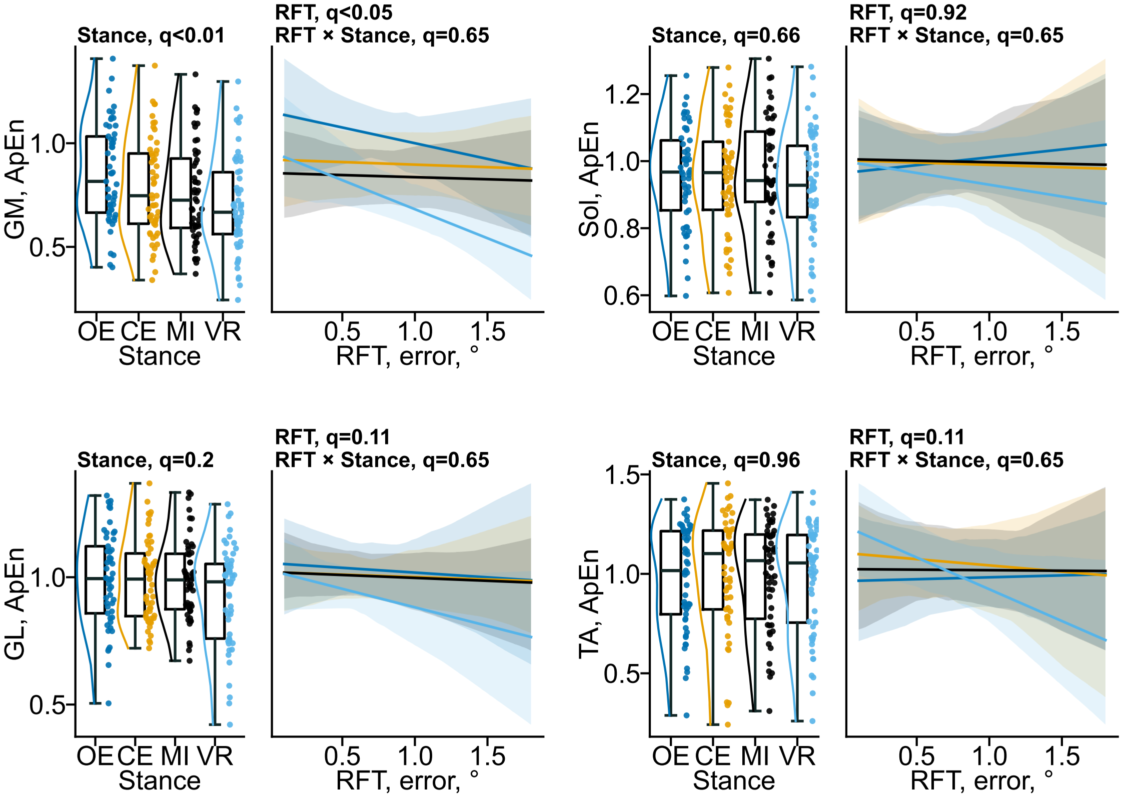
Stance, q<0.01
RFT, q<0.05
RFT × Stance, q=0.65
GM, ApEn
OE CE MI VR
Stance
RFT, error, °
Stance, q=0.66
RFT, q=0.92
RFT × Stance, q=0.65
Sol, ApEn
Stance, q=0.2
RFT, q=0.11
RFT × Stance, q=0.65
GL, ApEn
Stance, q=0.96
RFT, q=0.11
RFT × Stance, q=0.65
TA, ApEn
