# Supplementary material for: The Effects of Cognitive–Perceptual Style on the Multilevel Organization of Postural Control During Motor Imagery and Immersive Virtual Reality
Source: Behav Sci (Basel). 2026 Oct 15;16(10):1907. doi: 10.3390/bs16101907 (PMC13701233; doi:10.3390/bs16101907)
Supplement: Supplementary file 1 [file behavsci-16-01907-s001.zip › Supplementary File S2/Supplementary File S2.5 (m=3, r=0.2).pdf]

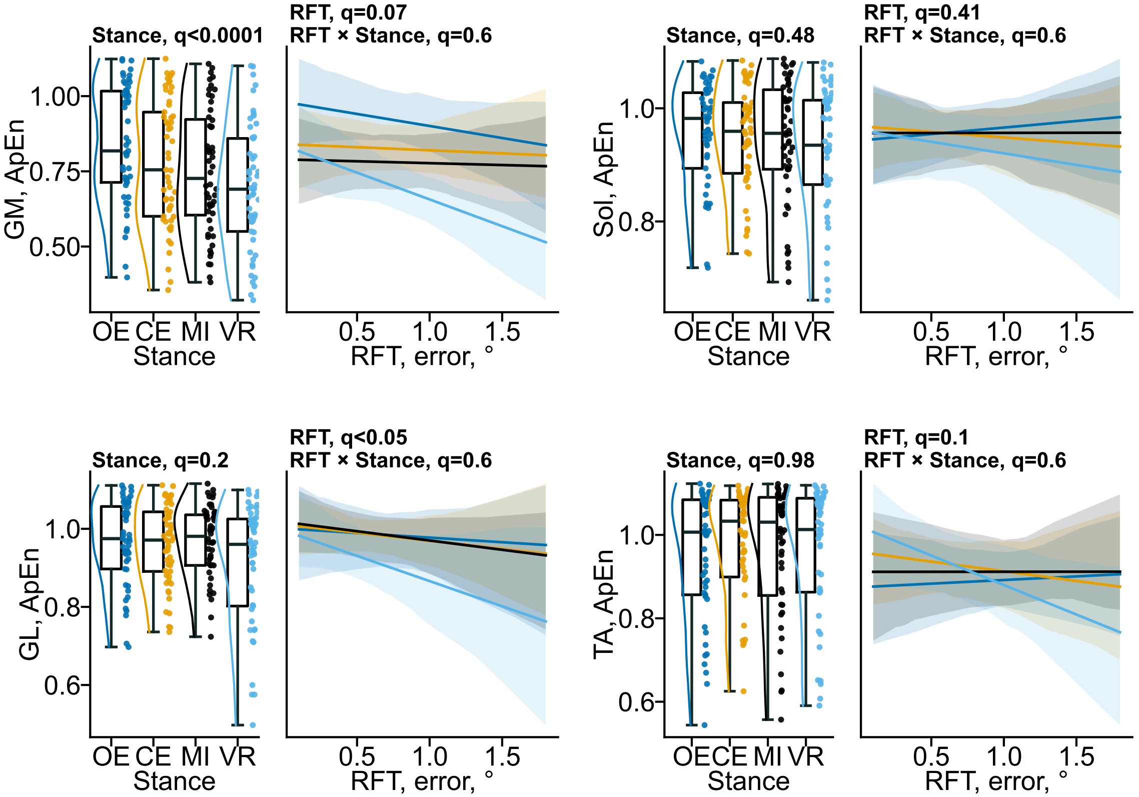
Stance, q<0.0001
RFT, q=0.07
RFT × Stance, q=0.6
Stance, q=0.48
RFT, q=0.41
RFT × Stance, q=0.6
GM, ApEn
Sol, ApEn
1.00
0.75
0.50
1.0
0.8
OE CE MI VR
Stance
0.5 1.0 1.5
RFT, error, °
Stance, q=0.2
RFT, q<0.05
RFT × Stance, q=0.6
Stance, q=0.98
RFT, q=0.1
RFT × Stance, q=0.6
GL, ApEn
TA, ApEn
1.0
0.8
0.6
