# Supplementary material for: The Effects of Cognitive–Perceptual Style on the Multilevel Organization of Postural Control During Motor Imagery and Immersive Virtual Reality
Source: Behav Sci (Basel). 2026 Oct 15;16(10):1907. doi: 10.3390/bs16101907 (PMC13701233; doi:10.3390/bs16101907)
Supplement: Supplementary file 1 [file behavsci-16-01907-s001.zip › Supplementary File S2/Supplementary File S2.6 (m=3, r=0.15).pdf]

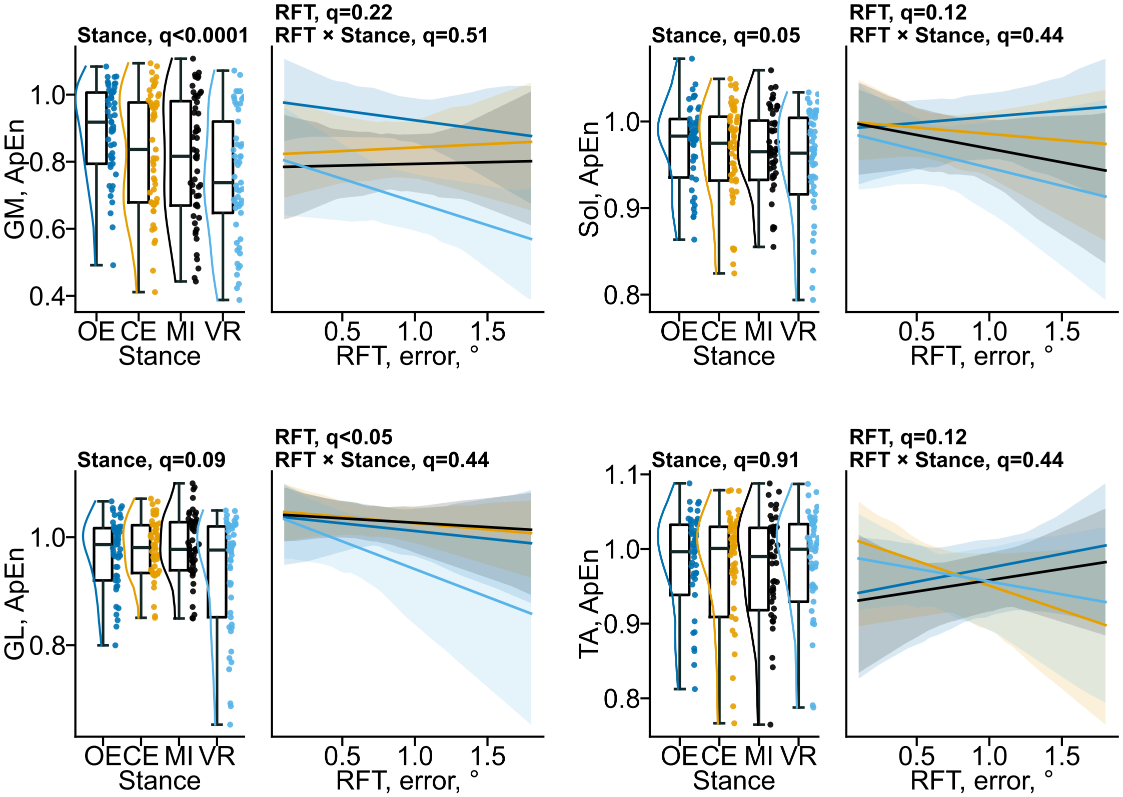

Stance, q<0.0001
RFT, q=0.22
RFT × Stance, q=0.51
GM, ApEn
1.0
0.8
0.6
0.4
OE CE MI VR
Stance
0.5 1.0 1.5
RFT, error, °
Stance, q=0.05
RFT, q=0.12
RFT × Stance, q=0.44
Sol, ApEn
1.0
0.9
0.8
OE CE MI VR
Stance
0.5 1.0 1.5
RFT, error, °
Stance, q=0.09
RFT, q<0.05
RFT × Stance, q=0.44
GL, ApEn
1.0
0.8
OE CE MI VR
Stance
0.5 1.0 1.5
RFT, error, °
Stance, q=0.91
RFT, q=0.12
RFT × Stance, q=0.44
TA, ApEn
1.1
1.0
0.9
0.8
OE CE MI VR
Stance
0.5 1.0 1.5
RFT, error, °
