# Supplementary material for: The Effects of Cognitive–Perceptual Style on the Multilevel Organization of Postural Control During Motor Imagery and Immersive Virtual Reality
Source: Behav Sci (Basel). 2026 Oct 15;16(10):1907. doi: 10.3390/bs16101907 (PMC13701233; doi:10.3390/bs16101907)
Supplement: Supplementary file 1 [file behavsci-16-01907-s001.zip › Supplementary File S2/Supplementary File S2.7 (m=3, r=0.25).pdf]

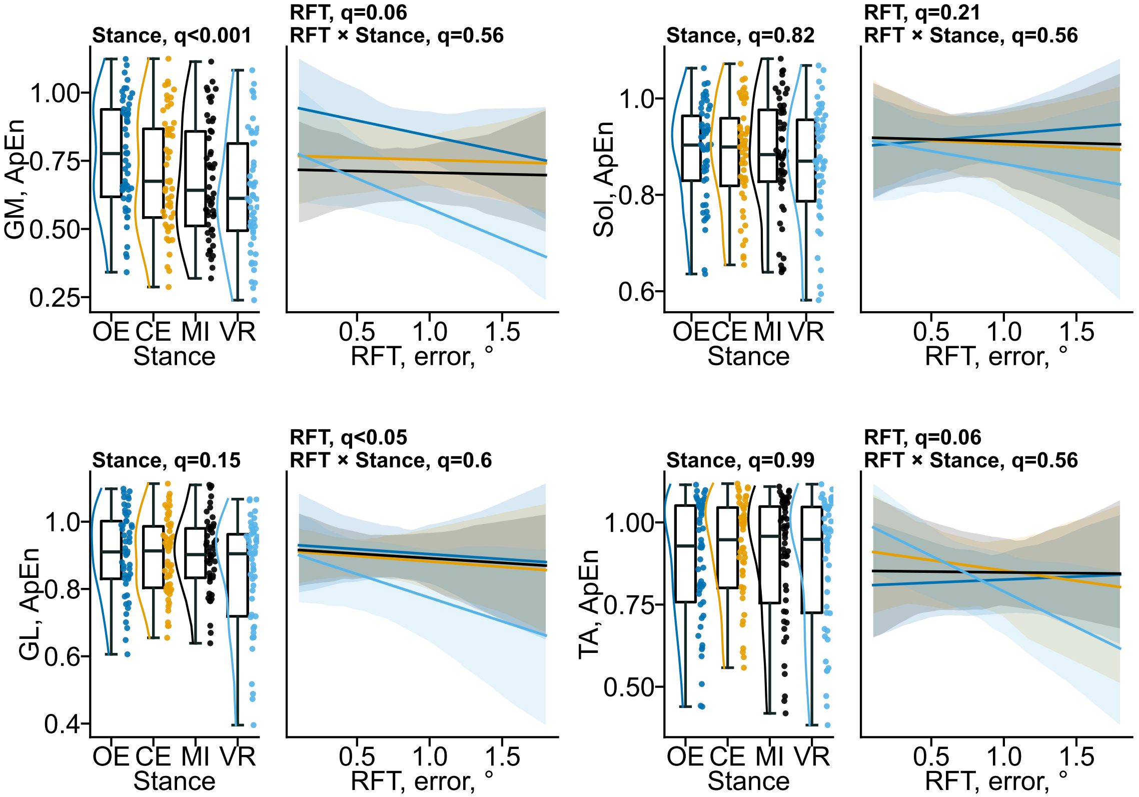
Stance, q<0.001
RFT, q=0.06
RFT × Stance, q=0.56
GM, ApEn
1.00
0.75
0.50
0.25
OE CE MI VR
Stance
0.5 1.0 1.5
RFT, error, °
Stance, q=0.82
RFT, q=0.21
RFT × Stance, q=0.56
Sol, ApEn
1.0
0.8
0.6
Stance, q=0.15
RFT, q<0.05
RFT × Stance, q=0.6
GL, ApEn
0.4
Stance, q=0.99
RFT, q=0.06
RFT × Stance, q=0.56
TA, ApEn
